# Selective killing of spinal cord neural stem cells impairs locomotor recovery in a mouse model of spinal cord injury

**DOI:** 10.1186/s12974-018-1085-9

**Published:** 2018-02-23

**Authors:** Melania Cusimano, Elena Brambilla, Alessia Capotondo, Donatella De Feo, Antonio Tomasso, Giancarlo Comi, Patrizia D’Adamo, Luca Muzio, Gianvito Martino

**Affiliations:** 1 Neuroimmunology Unit, Division of Neuroscience, Institute of Experimental Neurology (INSPE), San Raffaele Scientific Institute, Via Olgettina 58, 20132 Milan, Italy; 2 Department of Neurology, Institute of Experimental Neurology (INSPE), Vita Salute San Raffaele University, 20132 Milan, Italy; 3 Molecular Genetics of Intellectual Disabilities Unit, Division of Neuroscience at San Raffaele Scientific Institute, Milan, Italy

**Keywords:** Spinal cord injury, Endogenous neural stem cells, Neurotrophic factors, Inflammation

## Abstract

**Background:**

Spinal cord injury (SCI) is a devastating condition mainly deriving from a traumatic damage of the spinal cord (SC). Immune cells and endogenous SC-neural stem cells (SC-NSCs) play a critical role in wound healing processes, although both are ineffective to completely restore tissue functioning. The role of SC-NSCs in SCI and, in particular, whether such cells can interplay with the immune response are poorly investigated issues, although mechanisms governing such interactions might open new avenues to develop novel therapeutic approaches.

**Methods:**

We used two transgenic mouse lines to trace as well as to kill SC-NSCs in mice receiving SCI. We used Nestin CreERT2 mice to trace SC-NSCs descendants in the spinal cord of mice subjected to SCI. While mice carrying the suicide gene thymidine kinase (TK) along with the GFP reporter, under the control of the *Nestin* promoter regions (Nestin^TK^ mice) were used to label and selectively kill SC-NSCs.

**Results:**

We found that SC-NSCs are capable to self-activate after SCI. In addition, a significant worsening of clinical and pathological features of SCI was observed in the Nestin^TK^ mice, upon selective ablation of SC-NSCs before the injury induction. Finally, mice lacking in SC-NSCs and receiving SCI displayed reduced levels of different neurotrophic factors in the SC and significantly higher number of M1-like myeloid cells.

**Conclusion:**

Our data show that SC-NSCs undergo cell proliferation in response to traumatic spinal cord injury. Mice lacking SC-NSCs display overt microglia activation and exaggerate expression of pro-inflammatory cytokines. The absence of SC-NSCs impaired functional recovery as well as neuronal and oligodendrocyte cell survival. Collectively our data indicate that SC-NSCs can interact with microglia/macrophages modulating their activation/responses and that such interaction is importantly involved in mechanisms leading tissue recovery.

**Electronic supplementary material:**

The online version of this article (10.1186/s12974-018-1085-9) contains supplementary material, which is available to authorized users.

## Background

Spinal cord injury (SCI) is a chronic disease that generally affects young people, showing incidences that range from 10 to 900 per million worldwide [1]. The management of SCI requires impressive health care resources, and the clinical amelioration achieved so far can only modestly increase the quality of life in patients. Recently, neural stem cell (NSC)-based treatments have received a great deal of scientific interest. Accordingly, transplanted NSCs are neuroprotective because they secrete neurotrophic factors and offer bystander effects that sustain tissue recovery in several models of neurodegenerative disorders [2, 3]. This multifaceted therapeutic activity promotes the re-programming of the hostile microenvironment to a more instructive one, which eventually induces tissue repair [4]. Besides transplanted NSCs, also endogenous spinal cord NSCs (SC-NSCs) are critically involved in the central nervous system (CNS) recovery after SCI. Indeed, SC-NSCs self-activate after a traumatic injury and secrete neurotrophic factors that promote neuronal survival [5]. SC-NSCs are embedded in the ependymal cell layer, which is lining the central canal of the SC, arranged in specific germinal niches. Such niches show a different cyto-architecture, if compared with niches of the brain sub-ventricular zone (SVZ), [6, 7]. A key distinction is related to the position of proliferating cells within SC-confined niches. Indeed, dividing SC-NSCs are directly located in the ependymal layer rather than in the sub-ependymal one. Moreover, proliferating SC-NSCs typically self-renew themselves, rather than producing transit-amplifying progenitors. Their descendants remain within the ependymal layer and do not share any lineage relationship with sub-ependymal cells [8, 9]. In response to tissue damage, such as after SCI, SC-NSCs increase rates of cell proliferation, giving rise to astrocytes that contribute to the formation of the astroglial scar [5]. However, a fraction of SC-NSCs also express Olig-2 and acquire the cell morphology of immature oligodendrocytes that eventually migrate to the injury site. Later on, such cells differentiate in myelinating oligodendrocytes that finally enwrap axons [10, 11].

Similarly to other neuroinflammatory disorders, immune cells dominate the early post traumatic events occurring upon SCI and the prolonged secondary injury phase, which may last up to weeks or months. Indeed, the pathophysiology of SCI involves an initial injury which is followed by a sequela of secondary damages that includes many multifaceted pathological mechanisms. Among them, the inflammatory cascade arises from innate (neutrophils, macrophages, and microglia) and adaptive (B and T) immune responses. Such cells release inflammatory cytokines and chemokines, nitric oxide oxidants, proteases, and complement proteins [12]. About myeloid cells, microglia are rapidly activated in response to the SCI, while monocyte-derived macrophages enter the CNS few days after the trauma and their number generally peaks 7–10 days after SCI [13]. It is likely that either microglia or macrophages undergo activation acquiring a complex functional phenotype which involves the expression of pro-inflammatory as well as anti-inflammatory gene sets. The acquisition of such phenotypes is highly flexible and influenced by complex cascades of agonistic and antagonistic cues [14]. Infiltrating monocyte-derived macrophages are centrally involved in tissue repair processes, as shown in SCI mice receiving infusion of a supplemental amount of monocytes [15]. Similarly, the depletion of monocytes, but not of microglia, impairs recovery of mice upon SCI [16]. Infiltrating myeloid cells express CD11b, CD115, F4/80, Gr1, and activation markers such as CD11c and MHC-II. The selective ablation of CD11c in infiltrating myeloid cells affects recovery of SCI mice [16]. However, also activated microglia express CD11c at high levels [17] and considering that activated microglia and infiltrating macrophages could differentially modulate tissue repair, is not surprising that SCI mice transplanted with NSCs display a functional recovery and a remarkable reduction of CD11c^+^ cells [4]. In this study, we aimed at determining contribution of SC-NSCs to the functional recovery of mice receiving SCI. We selectively ablated SC-NSCs using transgenic mice that express the suicide thymidine kinase 1 (TK) gene under the control of *Nestin* regulatory regions. Our results show that SC-NSCs depletion causes a substantial reduction of growth factors in the injured tissue, increased demyelination and impaired locomotion recovery.

## Methods

### Study approval

Mice were maintained under pathogen-free conditions at San Raffaele Hospital mouse facility (Milan, Italy). All efforts were made to minimize animal suffering and to reduce the number of mice used, in accordance with the European Communities Council Directive of 24 November 1986 (86/609/EEC). All procedures involving animals were executed according to the guidelines of the Institutional Animal Care and Use Committee (protocol number: 622) of San Raffaele Scientific Institute, Milan.

### Spinal cord neural stem cells culture

SC-NSC cultures were raised from Nestin ^flox^GFP^flox^-TK mice according our published methods [18, 19]. Briefly, mice were deeply anesthetized by ketamine/xilazine and killed by cervical dislocation. SCs were removed and placed in chilled Hanks’ Buffered Salt Solution (HBSS) without Ca^2+^ and Mg2^+^, then cut into 1-mm^3^ pieces. Single-cell suspension was obtained by using Neural Dissociation Kit (P) (Miltenyi) according to the manufacturer’s instructions. Cells were cultured in NeuroCult® Proliferation Kit (Stem Cell Technologies). To profile cell growth curves, we plated 8000 cells/cm^2^ at each sub-culturing passage in untreated tissue culture flasks. After 2–3 days (time estimated to obtain the doubling of cells), neurospheres were harvested, mechanically dissociated, counted, and re-plated under the same culture conditions. For each experiment, we used SC-NSCs with less than 20 passages. We characterized SC-NSCs by flow cytometry as described [20, 21]. Briefly cells were stained with fluorophore-conjugated PE-α mouse and human SOX-2 (Miltenyi), rat-α alpha4 integrin (clone PS/2, Abcam), rat-α CD44 (clone IM7, BD Biosciences), or rat-α CXCR4 (clone 2B11/CXCR4, BD Biosciences) diluted in mouse FcR blocking reagent. Cells were labeled for 10 min then rinsed with PBS and re-suspended in PBS. Flow cytometry was done on a Cyan-ADP (Dako Cytomation) or FACSCanto® II flow cytometer (BD) using FlowJo (Treestar) software. P2 bulk cultures obtained from Nestin ^flox^GFP^flox^-TK mice were sorted on the basis of their GFP expression levels using MoFlo XDP, Cell Sorter (Beckman Coulter).

Immunofluorescence on SC-NSC cultures was done according our published methods [22]. Briefly cells were fixed with 4% paraformaldehyde 10′ at room temperature, then rinsed three times with PBS, and then incubated for 60 min with a blocking solution [PBS, 10% normal goat serum (NGS, Sigma), 0.1% albumin bovine serum (BSA, Sigma)] to avoid a-specific binding of antibodies. For intracellular staining, we added 0.1% Triton X-100 in blocking solution. Cells were incubated with the appropriate primary antibody for 2 h. Cells were then washed in PBS and then incubated for 45 min with fluorescent secondary antibodies. The nuclei were stained with 4, 6-diamine-2-fenilindole (1 μg/ml, DAPI, Roche). Cells were then washed and mounted with Fluorescent mounting medium (Dako). The following antibodies were used: Rb-α GFAP (Dako), mouse-α O4 (clone 81, Millipore), and mouse-α NeuN (clone A60, Millipore). Imaging was done using Leica SP5 confocal microscope equipped with LasX software.

### Transgenic mice and ganciclovir regimen

Selective ablation of SC-NSCs was done in Nestin^TK^ mice that were originated from Nestin ^flox^GFP^flox^-TK mice [19] in which GFP excision was done using CMV-Cre mouse line [23]. Nestin^TK^ mice express thymidine kinase 1 (TK) under the control of the second intron enhancer of *Nestin* [24]. Mice received 100 mg/kg/day of ganciclovir (GCV) dissolved in distilled water by subcutaneously implanted osmotic minipumps (ALZET® Model 2002, DURECT Corporation) [19]. After 14 days, pumps were replaced with new ones, thus allowing 28 days of continuous treatment. Controls were raised using WT liters with or without GCV regimen. To evaluate the ablation of SC-NSCs, 5-Bromo-2′-deoxyuridine (BrdU, 1 mg/ml dissolved in 1% sucrose) was administered in the drinking water continuously for a month. Depending on the experimental setting, cell proliferation in SCI mice was also evaluated by BrdU intraperitoneal injections (100 mg/kg). Cell tracing was done using mice carrying the Nestin^CreERT2^ allele [25] that were crossed with the reporter mouse line Rosa 26YFP [26]. Double transgenic mice were supplemented with Tamoxifen (Sigma) for 5 days, washed out for further 5 days, and finally subjected to SCI.

### Contusion spinal cord injury

Twelve-week male mice (22–25 g) were anesthetized with ketamine/xylazine and a laminectomy was performed in thoracic spinal cord at the level of the T12 vertebra. Contusions were generated applying 75 k-dynes with an Infinite Horizons Impactor (*Precision systems and Instrumentation*), [16]. As postoperative care, we administered enrofloxacin (Baytril, Bayer; 2.5 mg/kg, subcutaneously) once daily for 2 weeks. Urine was expelled by manual abdominal pressure twice daily for 1 week and then once daily for the duration of the experiment. The recovery of locomotor performance was evaluated using the Basso Mouse Scale (BMS), as previously described [16]. Briefly, mice were observed individually for 4 min each in an open field by three investigators blinded to surgery procedures and genotype. Hind limb motor function was recorded and scored according to the BMS guidelines. For statistical analyses we calculated means of left and right hind limb scores for each animal. Catwalk XT Gait Analysis System (Noldus Information Technology, Asheville, NC, USA) was used to assess walk ability of mice. Mice were allowed to freely ambulate along an illuminated glass plate within a confined corridor (L 50 cm × W 8 cm) in a darkened room and footprints were recorded with a high-speed camera for following analyses with CatWalk XT 10.0 software (Noldus).

### Tissue processing and histopathology

Mice were deeply anesthetized with ketamine/xilazine and transcardially perfused with 4% paraformaldehyde. Spinal cords were removed and post-fixed in the same solution for 12 h at 4 °C and then washed with PBS. The dissected spinal cords were cryoprotected for 24 h in 30% sucrose (Sigma) at 4 °C, then spinal cords were embedded in Tissue Tek (EM sciences) and snap frozen with liquid nitrogen. Frozen spinal cords were axially sectioned to generate 10-μm-thick coronal sections. A total of 7 mm of each cord segment (centered on the impact site) was cut, collected in serial sections (100 μm apart) and stored at − 80 °C. Four spinal cord segment-informative tissue slides (each containing *n* = 18 spinal cord 10 μm tick axial sections, for a total of *n* = 72 sections for each animal) were processed for qualitative and quantitative histopathology by immunohistochemistry with appropriate primary antibodies. Anti-rat, anti-mouse, anti-goat, and anti-rabbit fluorophore (Alexafluor 488, 546; Molecular Probes) or biotin (Amersham biosciences)-conjugated secondary antibodies were used to detect the signal. Nuclei were stained with 4-6-diamidino-2-phenylindole (DAPI; Roche). The following primary antibodies and working concentrations were used: Rabbit α-GFAP 1:500 (dako), rat-α BrdU 1:500 (Abcam), mouse-α NeuN 1:200 (Millipore), rabbit α-Olig 2 1:200 (Abcam), chicken α-GFP 1:500 (Millipore), rabbit α Iba1 1:400 (Wako), and mouse-α F4/80 1:100 (Abcam). A Leica M4000B microscope equipped with Neurolucida (Version 8.0 Software, Microbrightfield) was used to calculate the volumes of the lesion, the volumes of demyelination, as well as the cell numbers by stereology. Fluorescence analysis was performed using the confocal microscope Leica SP5 equipped with a × 40 objective. The lesion volumes were calculated as contours on GFAP-labeled spinal cord segment-representative 10-μm-thick axial sections (100 μm apart). Calculations were made imaging five to eight mice per group with × 5 or × 10 magnifications and lesion volumes were expressed in cubic millimeter. The lesion volume contours were traced at the boundaries between GFAP-positive and GFAP-negative regions according published methods [27]. Demyelination volumes were calculated as contours on Luxol-Fast blue-stained spinal cord segment-representative 10-μm-thick axial sections accordingly our published method [4]. Calculations were made imaging four to five mice per group with × 2.5 or × 10 magnifications and lesion volumes were expressed in cubic millimeter.

### Quantification of cell number

The number of GFP^+^/BrdU^+^ cells was calculated in correspondence of the closest spared central canal to lesion epicenter for up to 2 mm both rostrally and caudally (*n* = 3 mice per group). Olig2^+^ and NeuN^+^ cells were counted from the closest spared cross-section to lesion epicenter for up to 4 mm, both rostrally and caudally. The number of Olig2^+^ cells was calculated in correspondence of the dorsal funiculus in a trapezoidal area (0.08 mm^2^) per section (*n* = 3 mice per group). The number of NeuN^+^ cells was calculated in correspondence of four different regions (lamina X, ventral horn, dorsal horn and lateral gray matter) for a total area of 0.1 mm^2^ per section (*n* = 3 mice per group).

### RNA isolation, reverse transcription, and quantitative real-time polymerase chain reaction

Segments encompassing the thoracic spinal cord T11-T13 region were rapidly sampled and lysed for total RNA extraction using RNeasy® Mini kit (Qiagen) kit according to the manufacturer’s recommendations including DNase digestion. cDNA synthesis was performed by using ThermoScriptTM RT-PCR System (Invitrogen) and Random Hexamer (Invitrogen). Real-time PCR was done using the following TaqMan® probes (Applied Biosystems):

**Table:** 

Gene	Cod AB
GAPDH	Mm99999915_g1
IGF1	Mm00439561_m1
LIF	Mm00434761_m1
CNTF	Mm00446373_m1
Vegfa	Mm00437304_m1
TNF	Mm00443258_m1
IL1b	Mm01336189_m1
iNos(NOS2)	Mm00440502_m1
IL6	Mm00446190_m1

### Ex vivo cytofluorimetric analyses of immune cells

Mice subjected to spinal cord injury were killed by an overdose of anesthetic and their spinal cords, processed for flow cytometry after saline transcardial perfusion. The lesioned cord segments extending between T11 and T13 were homogenized and reduced to single-cell suspensions by Neural Tissue Dissociation Kit (P) (Miltenyi Biotec) according to the manufacturer’s instructions. Cell suspension was filtered through a 70-μm strainer, centrifuged over a 30% discontinuous Percoll Gradient (GE Healthcare) and myelin debris removed from the surface (modified from Cardona et al., [28]). Cells were labeled with Zombie Aqua™ die (Biolegend), then incubated with 5% FBS, 1%BSA, and 5 mg/ml rat anti-mouse Fc III/II receptor (CD16/CD32) blocking antibodies (BD), and then stained using the following monoclonal antibodies: CD45-Pb (Biolegend), CD11b-PeCy7 (BD), Ly6C-APCCy7 (BD), Ly6G-PerCP Cy5.5 (BD), and CD11c-APC (ebioscence). Cells were analyzed by LSR Fortessa (Beckton Dickinson) and data analyses by FlowJo (Treestar) software.

### Statistics

Sample size for each experiment was estimated at the beginning of the study with the PS power and sample size calculator software (version 3.0.43) based on variance data obtained in previous experiments [4]. Data are expressed as the mean ± standard error of the mean (S.E.M.) or mean ± standard deviation (S.D.) of independent experiments. Normality was assessed in each experiment by applying either Kolmogorov–Smirnov test (with Dallas–Wilkinson–Lille for *P* value) or D’ Agostino & Pearson omnibus normality test. Comparisons were made using the unpaired *t* test and one-way or two-way analysis of variance (ANOVA) tests, followed by Tukey’s multiple comparison test. Nonparametric data have been compared using Mann Whitney test or Kruskal-Wallis test followed by Dunn’s multiple comparison test. Statistical tests were carried out using PRISM5.01 (GraphPad Software, La Jolla, CA, USA). Value less than 0.05 was considered statistically significant.

## Results

### Central canal-derived cells from Nestin floxGFPflox-TK mice generate neurospheres

It has been described that ependymal cells lining the central canal of the SC retain features of neural stem cells [5, 11, 29]. We first evaluated whether Nestin ^flox^GFP^flox^-TK transgenic mice can be used to manipulate these cells in a SCI model [19]. This transgenic mouse line was successfully used to study SVZ-confined neural stem cells [19], while these mice were never investigated in the SC. We started to score GFP expressing cells in the SC of adult Nestin ^flox^GFP^flox^-TK mice. The reporter GFP was expressed in the ependymal cells of the central canal, while not expressed in parenchymal cells and in particular in endothelial cells that in the brain are known to be positive for Nestin. This result confirmed previous observation showing that the use of the II intron enhancer of the *Nestin* promoter restricts the transgene expression to NSCs [30]. Furthermore, none of the GFP^+^ cells co-expressed the GFAP marker in the SC (Additional file 1: Figure S1A), while, some GFP^+^ ependymal cells were positive for Id1, which is a dominant-negative helix-loop-helix transcriptional regulator that identifies self-renewing NSCs of the SVZ (Additional file 1: Figure S1B), [31].

**Fig. 1:**
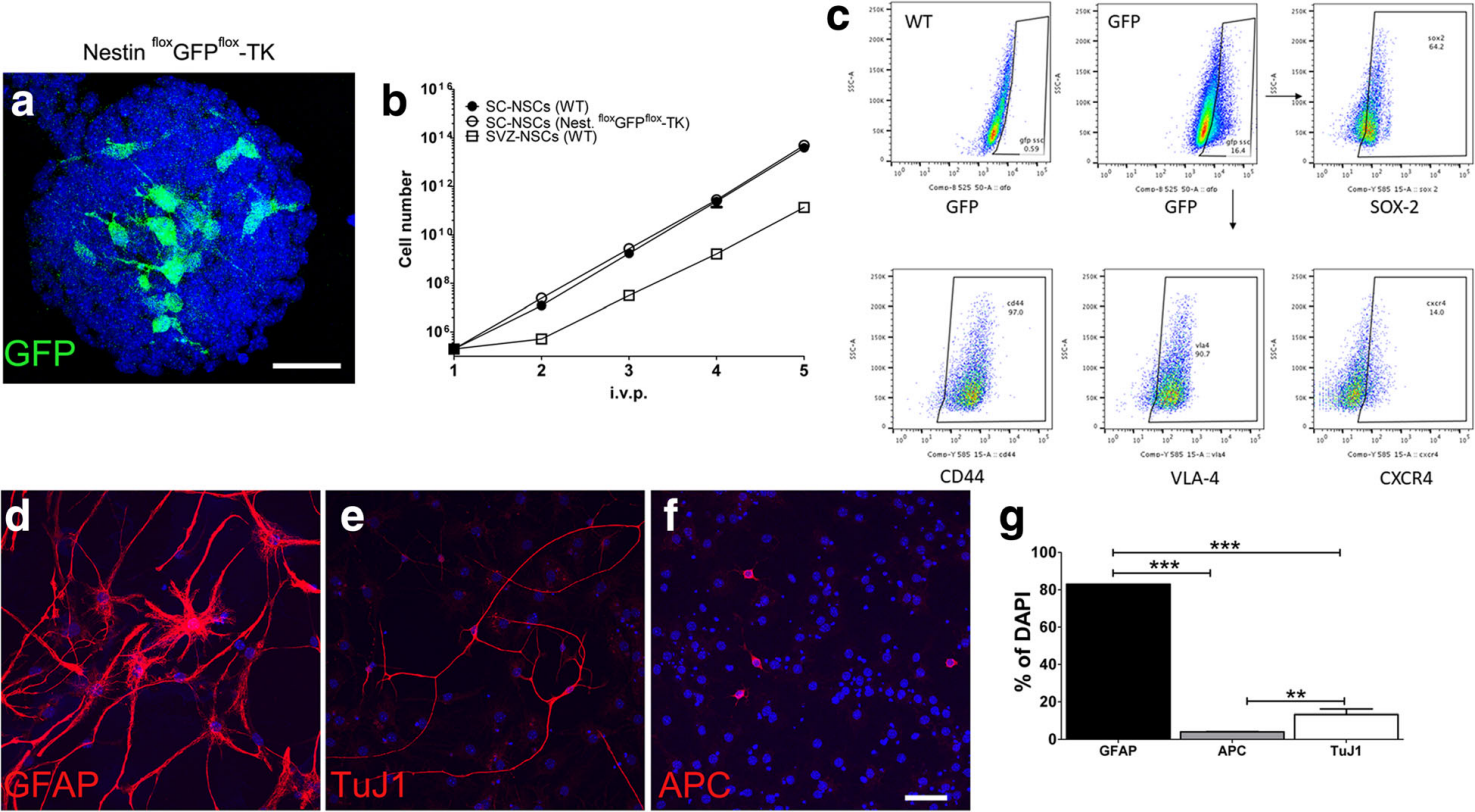
Ependymal cells lining the spinal cord central canal display neural stem cells properties in vitro. **a** Representative confocal image of a primary neurosphere raised from Nestin ^flox^GFP^flox^-TK mice. Nestin^+^ (GFP) cells are embedded inside the sphere. **b** Growth curves of NSCs derived from the SC of WT (black dots) and of Nestin ^flox^GFP^flox^-TK (white dots) mice. SVZ-derived neurospheres cultures were raised from WT mice and used as control (white square), *n* = 3 for each group. **c** Flow cytometry analysis of GFP^+^ cells labeled for SOX-2, CD44, VLA4, and CXCR4. Cells were obtained from in P2-dissociated neurospheres of SC of Nestin ^flox^GFP^flox^-TK mice. The GFP gate was established using cultures from WT SCs (*n* = 3). **d**–**g** Representative confocal images showing SC-NSCs from Nestin^flox^GFP^flox^-TK mice plated on Petri dishes in the absence of growth factors and labeled for GFAP (**d**), TuJ1 (**e**), and APC (**f**). Percentages are calculated on the total number of nuclei (DAPI) and quantifications are shown in panel (**g**). Data are collected from one representative experiment that has been replicated two times. One-way ANOVA followed by Tukey multiple comparisons post-test has been used to analyze data of panel **g**. ** *p* = 0.0052, ***p < 0.001. Scale bar 30 μm

We next raised NSCs cultures from the SC of Nestin ^flox^GFP^flox^-TK mice to further demonstrate that SC-restricted GFP^+^ cells possess features of bona fide NSCs. We included in our experiments a functional control represented by SVZ-derived NSC cultures [32]. Dissociated SCs from adult Nestin ^flox^GFP^flox^-TK mice gave raise to primary neurospheres that were characterized by the presence of GFP^+^ cells in virtually all spheres we assayed (Fig. 1a). We next assayed self-renewal potentials of SC-NSCs comparing their ability to growth in vitro as normally observed in SVZ-derived NSC cultures. We found that both NSCs cultures displayed similar growth curves (Fig. 1b). In the SVZ, Nestin is transiently expressed by activated type-B1 cells, while quiescent B1 cells are negative and levels of this marker generally drop when cells undergo to cell differentiation [33–35]. Because we initially examined cultures that were composed by a mixed cell population containing either GFP^+^ or GFP^−^ cells, we next sorted GFP^+^ cells from Nestin ^flox^GFP^flox^-TK-derived SC cultures to generate neurospheres. Dissociated cells from SCs of Nestin ^flox^GFP^flox^-TK mice were expanded in vitro for two passages, then GFP^+^ cells were sorted by flow cytometry (Additional file 2: Figure S2B). GFP^+^ cells were plated at the concentration of 8000/cm^2^, left to grow in standard NSC medium and examined for the generation of spheres. Three days after plating several cells started to generate small spheres that reached the morphology and the size of classical SVZ-derived NSCs neurospheres after 7 days (Additional file 2: Figure S2C, D). We next examined some markers that are commonly expressed by SVZ-derived NSCs cultures [18]. We observed that around 64% of the GFP^+^ cells derived from SCs of Nestin ^flox^GFP^flox^-TK mice expressed SOX2. CD44 and VLA4 were expressed by the vast majority of these GFP^+^ cells, while CXCR4 expression was confined in a small percentage of them (Fig. 1c). Dissociated SC-NSC neurospheres were plated in differentiation medium and labeled for GFAP, TuJ1, and APC. We observed that SC-NSCs from Nestin ^flox^GFP^flox^-TK mice can differentiate in astrocytes, neurons, and oligodendrocytes (Fig. 1d–g). Thus, in agreement with previously published results, we observed that GFP^+^ ependymal cell cultures from Nestin ^flox^GFP^flox^-TK mice have bona fide features of NSCs [10].

### Traumatic spinal cord injury triggers endogenous SC-NSC proliferation

**Fig. 2:**
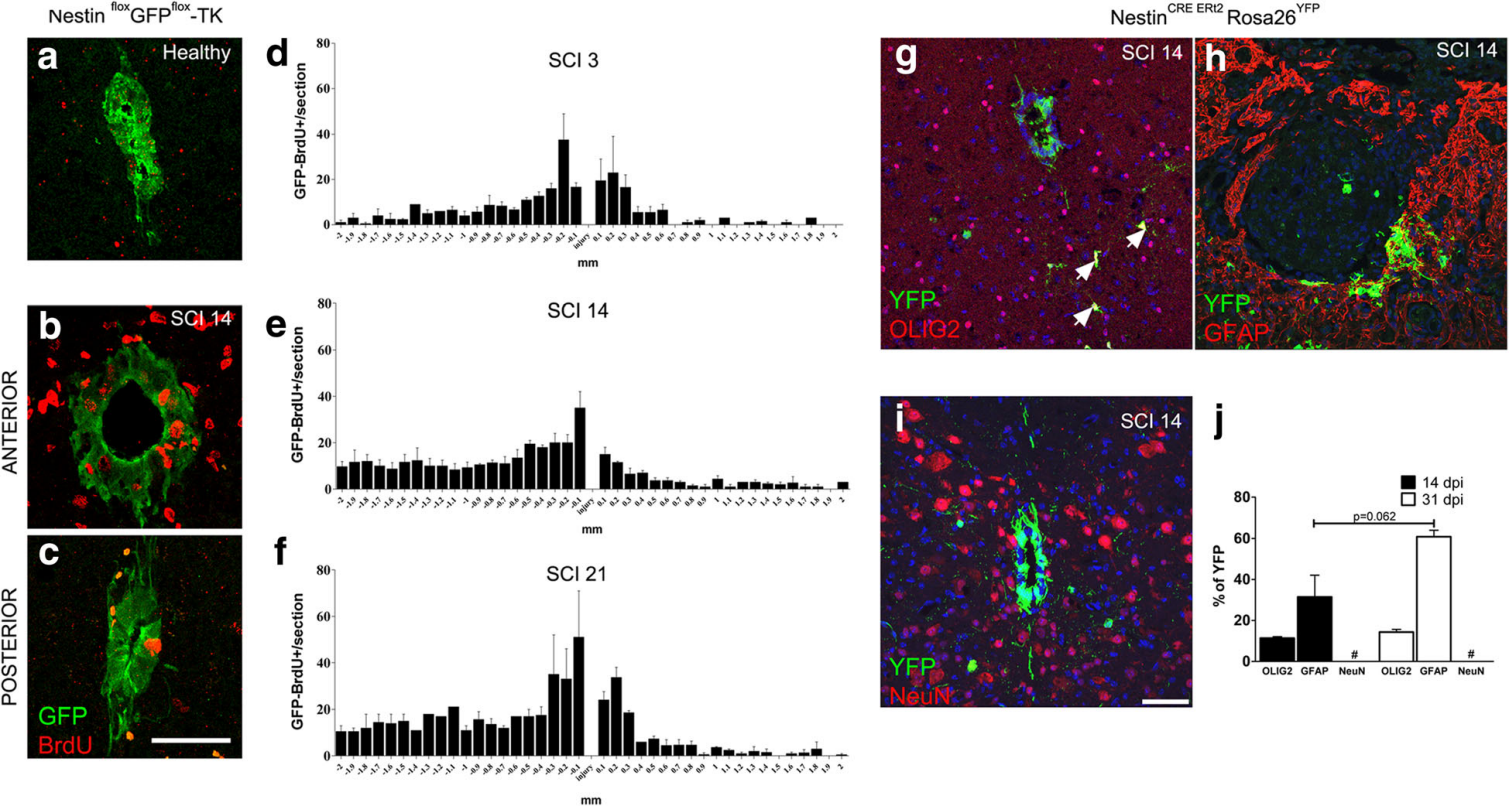
SC-NSC activation and differentiation upon SCI. **a** Representative confocal images showing the central canal of healthy Nestin ^flox^GFP^flox^-TK mice labeled for GFP and BrdU. **b**, **c** Segments proximal (**b**) and distal (**c**) to the lesion epicenter from a Nestin ^flox^GFP^flox^-TK mouse sampled 14 days after SCI. **d**–**f** Quantifications of GFP^+^BrdU^+^ cells (means ± S.E.M) within the ependymal layer of the central canal in Nestin ^flox^GFP^flox^-TK mice sacrificed 3, 14, and 21 days after SCI (*n* = 3 mice/time point). **g**–**j** Nestin^CreERT2^Rosa 26^YFP^ mice were injected with TAM for 5 days and, following a washout of further 5 days, subjected to injury. Confocal images of OLIG2/YFP (arrows indicate double positive cells), GFAP^+^YFP^+^, and NeuN^+^YFP^+^ are provide in **g**–**i**. Quantifications (means ± S.E.M.) are plotted on **j**, (*n* = 3 for each group). Two-way ANOVA, followed by Bonferroni’s multiple comparison test has been used to analyze data of **j**. Scale bar 30 μm

We next investigated cell proliferation of GFP^+^ cells in the intact and above all in the injured SC [11]. Cell proliferation was assayed in mice receiving a severe contusion of the SC that was obtained using IH impactor at T12 thoracic level. After the injury, Nestin ^flox^GFP^flox^-TK mice received daily injections of BrdU and proliferating cells were scored 3, 14, and 21 days after the contusion. As expected, unaffected Nestin ^flox^GFP^flox^-TK mice displayed few GFP^+^BrdU^+^ cells in the SC (Fig. 2a), while their numbers were substantially increased upon SCI (Fig. 2b, c). Most of the GFP^+^BrdU^+^ cells was located anteriorly to the site of the injury, peaking in a region that was positioned 0.1–0.2 mm far for the lesion site (Fig. 2d–f). These results confirm and extend previously published data, showing increased BrdU uptake in the SC of SCI mice [36]. However, our data showed that cell proliferation persisted in regions flanking the site of the injury for more than 2 weeks (Fig. 2f), [11]. We next performed a fate map experiment tracing ependymal cells descendants using a transgenic mouse line in mice that carry the Nestin^CreERT2^ allele [25]. Nestin^CreERT2^ mice were crossed with Rosa26^YFP^ reporter mice [26], injected with Tamoxifen for 5 days, washed out for further 5 days, and finally subjected to SCI. Tracing of ependymal descendants was done 14 and 31 days after the induction of the injury. Sections were labeled for YFP and markers of oligodendrocytes precursors, astrocytes, and neurons (Fig. 2g–i). At both time points, most of the YFP^+^ cells co-expressed GFAP, a subset of them was Olig2^+^, while NeuN^+^YFP^+^ cells were never detected (Fig. 2j). These results show that SC-NSC descendants can give rise to oligodendrocytes precursors and astrocytes in response to tissue damage.

### Spontaneous functional recovery is impaired in GCV-Nestin^TK^ mice

We next genetically ablated SC-NSC to investigate their functional role in healing processes that spontaneously occur after SCI. We crossed Nestin ^flox^GFP^flox^-TK mice with mice carrying the CMV-Cre allele that ubiquitously express the Cre recombinase [23]. We successfully generated Nestin^TK^ mice in which the loxP- GFP cassette was excised, allowing mice to express a functional TK gene. We proved the efficiency of SC-NSC ablation, treating Nestin^TK^ mice along with their wild type (WT) littermates with GCV for 28 days. Mice received BrdU, and SC sections were scored for Vimentin (VIM) and BrdU to demonstrate the effective ablation of ependymal cells. We observed a substantial reduction of VIM^+^ cells in ependymal layer of Nestin^TK^ mice. Accordingly, the number of ependymal BrdU^+^ cells was severely diminished in GCV-Nestin^TK^ mice (Additional file 3: Figure S3A–C).

**Fig. 3:**
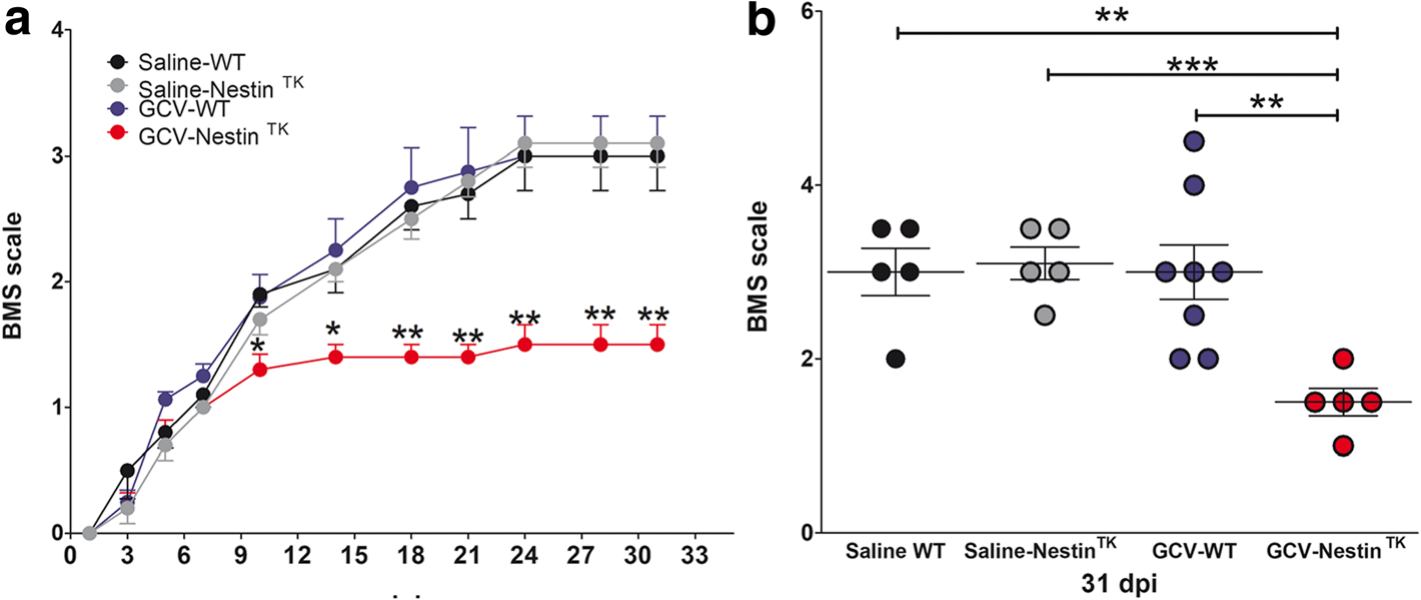
SC-NSCs are involved in the spontaneous process of functional recovery form spinal cord injury. Histogram in **a**, shows locomotor functions of saline control mice (black dots, *n* = 5), saline Nestin^TK^ mice (gray dots, *n* = 5), GCV-WT mice (blued dots, *n* = 8), and GCV-Nestin^TK^ mice (red dots, *n* = 5). Data are represented as Basso Mouse Scale (± S.E.M.) mean values of the locomotor score of each group. **b** Basso Mouse Scale (± S.E.M.) mean values of the locomotor score of individual mice on day 31 (each dot represent a single animal). Data derived from one representative experiment that has been replicated two times. Statistical analysis in **a** has been done comparing at each time point GCV-WT and GCV-Nestin^TK^ mice. Day 10, *p* = 0.044; day 14, *p* = 0.026; day 18, *p* = 0.007; day 21, *p* = 0.008; day 24, *p* = 0.0044; day 28, *p* = 0.0045; day 31, *p* = 0.0044. Results in **b** have been assayed by repeated measures ANOVA followed by Bonferroni’s multiple comparison test. **p = 0.0045 for GCV versus Nestin^TK^ + GCV; *p* = 0.0015 for Nestin^TK^ + GCV versus untreated; *p* = 0.0002 for Nestin^TK^ + GCV versus Nestin^TK^

We next subjected GCV-Nestin^TK^ and GCV-WT mice to SCI. We included in our experimental setting two additional groups of mice, which were composed by Nestin^TK^ and WT mice receiving saline, instead of GCV. Mice were scored for neurological functions until day 31 using the BMS score, which measures hind limb motor ability in an open field arena [37]. GCV-Nestin^TK^ mice, lacking the SC-NSCs, exhibited an impaired recovery, when compared to GCV-WT mice. Comparing BNS curves, we observed that differences in motor limb ability started to become statistically significant on day 10 and remained significant until the end of the experiment, at day 31 (Fig. 3a, b). We next induced SCI on further GCV-WT and GCV-Nestin^TK^ mice to evaluate their locomotion abilities using the Catwalk XT gait analysis [38]. Mice were collected at day 18 upon SCI and scored with the BMS. GCV-Nestin^TK^ mice displayed a significant worsening of scores when compare to GCV-WT mice (Additional file 4: Figure S4A). Mice were next assayed with the Catwalk to confirm the general impairment occurring to the GCV-Nestin^TK^ group. Anterior paws prints did not show any alteration in both experimental groups, while posterior paws prints were hardly detectable on the glass walkway. However, examining movie files, we observed that GCV-Nestin^TK^ mice showed a general worsening of locomotion that was featured by a lack of both hind leg prints due to an almost complete paralysis of the legs. On the other hand, GCV-WT mice showed a major rescue charged to the RH paw and partially to the LH paw (Additional files 5 and 6 show two representative animals from both groups). Accordingly, step timing views of two representative mice from each group, confirmed the substantial impairment of locomotion in the GCV-Nestin^TK^ mouse (Additional file 4: Figure S4 B, C).

**Fig. 4:**
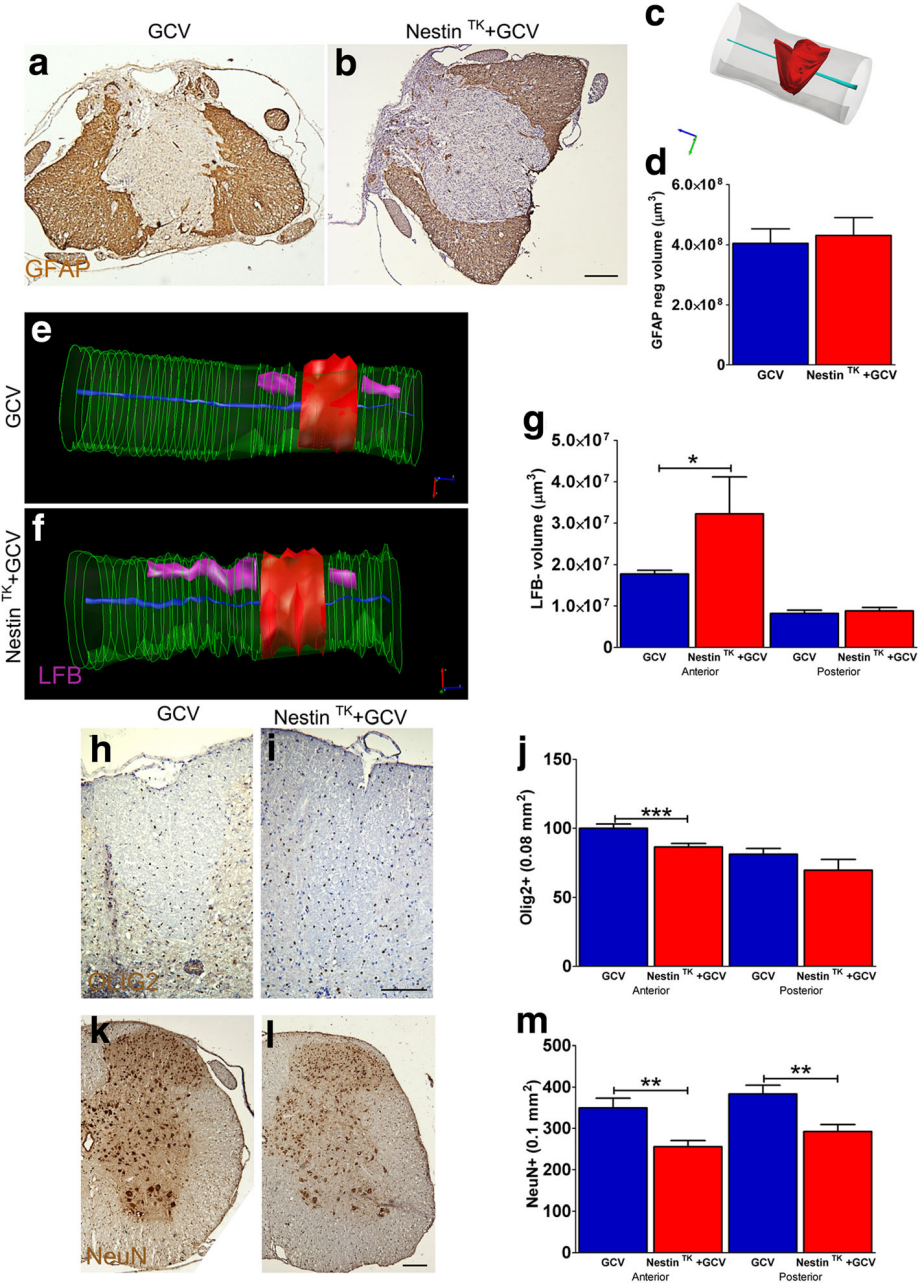
GCV-Nestin^TK^ mice show enhanced tissue damage after SCI. **a**–**d** Stereological quantification of the lesion volume as GFAP^−^ tissue injury in GCV-Nestin^TK^ and GCV-WT mice. **a**, **b** Representative axial images of GFAP-labeled sections from GCV-WT (**a**) and GCV-Nestin^TK^ (**b**) mice. In the 3D rendering **c**, the solid light blue is the central canal and the solid red is the volume negative for GFAP. Quantifications (means ± S.E.M.) are shown in **d** (*n* = 5–8 per group). **e**–**g** Stereological quantification of demyelination by Luxol fast blue (LFB) in GCV-Nestin^TK^ and GCV-WT mice, (**e**, **f** violet solid, *n* = 4–5 per group). Quantifications of the demyelination volumes (means ± S.E.M.) are shown in **g**, *p = 0.023. **h**–**i** Olig2^+^ *p* = 0.0005. **k**–**l**, NeuN^+^ cells (**p = 0.0058 anterior and **p = 0.0052 posterior) by immunohistochemistry in GCV-Nestin^TK^ and GCV-7WT mice. Quantifications (means ± S.E.M., *n* = 3 per group), performed and day 31, are provided in **j**, **m**. One-way ANOVA followed by Bonferroni’s multiple comparison test has been used to analyze data of **g**, **j**, and **m**. Scale bar 100 μm

To find out the mechanisms underlying the functional worsening of GCV-Nestin^TK^ mice, we first evaluated pathological features of mice at day 31. Since BMS scores of both Nestin^TK^ and WT mice receiving SCI and the saline treatment substantially overlapped, we only performed histological assessments on GCV-Nestin^TK^ and GCV-WT mice. We stereologically calculated the volumes of the glial scar, scoring the distribution of GFAP-positive and GFAP-negative areas around the site of the injury. GCV-Nestin^TK^ and GCV-WT mice displayed similar scar volumes, indicating that the lesion volume was not affected by SC-NSC ablation (Fig. 4a–b). On the other hand, demyelination was significantly increased in regions located anteriorly to the site of the injury in GCV-Nestin^TK^ mice (Fig. 4e–g). Parallel to demyelination, we also observed a significant reduction of Olig2^+^ progenitors in GCV-Nestin^TK^ mice (Fig. 4h–j). Finally, we noticed that the depletion of SC-NSCs caused a significant reduction of neurons in regions flanking the site of the injury. Interestingly, such alteration included the ventral horn and affected motor neurons, (Fig. 4k–m). Altogether, these data suggest that the functional worsening of GCV-Nestin^TK^ mice subjected to SCI parallels with a general reduction of oligodendrocytes and neurons.

### Loss of neurotrophic factors affects GCV-Nestin^TK^ mice

**Fig. 5:**
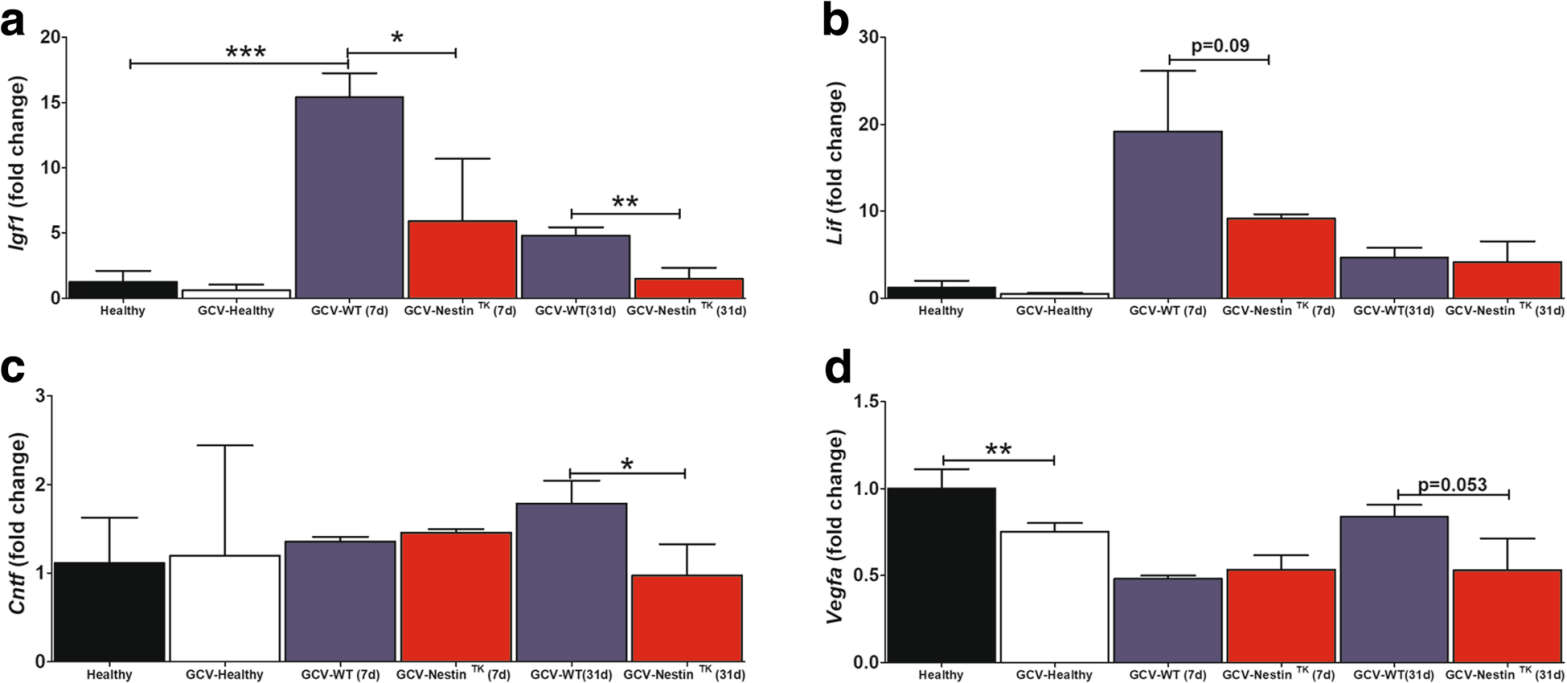
Downregulation of neurotrophic factors in GCV-Nestin^TK^ mice. Real-time PCR for *Igf1* (**a**), *Lif* (**b**), *CNTF* (**c**), and *Vegfa* (**d**) performed on total RNA extracts derived from T11–T13 spinal cord segments. Explants were collected at days 7 and 31. Values indicate the mean fold changes ± S.D. (*n* = 3–6 for each group) calculated on the expression levels measured in healthy mice (black bars). Comparisons were done using the *t* Student test: *Igf1*:***p = 0.0001, *p = 0.03, and *p* = 0.006; *Cntf*, *p* = 0.032; *Vegfa*, *p* = 0.008

Besides cell replacement, endogenous SC-NSCs support wound healing through the secretion of a complex array of neurotrophic factors, which directly or indirectly enhances neuronal survival [5]. Among such factors, SC-NSC-derived astrocytes secrete *Igf1* and *Cntf*, which play a crucial role in supporting neuronal survival after SCI [5]. We next investigated mechanisms regulating the lack of clinical recovery observed in GCV-Nestin^TK^ mice measuring the expression levels of such growth factors in a region encompassing T11–T13 spinal segments from SCs sampled at days 7 and 31. We also included in our analysis two genes that encode for *Lif* and *Vegfa*, which are two growth factors released by NSCs that upon transplantation mediate neuroprotective effects in animal models of multiple sclerosis and brain ischemia, respectively [39, 40]. *Igf1* expression levels were significantly dampened in GCV-Nestin^TK^ mice when compared to levels measured in GCV-WT litters at both days 7 and at 31 (Fig. 5). *Lif* mRNA levels were reduced at day 7 in GCV-Nestin^TK^ mice, although such reduction was a trend that did not reach the statistical significance (Fig. 5b). The inhibition of the SC-NSC cell proliferation observed in FoxJ1-rasless mice affects SCI outcomes and induces a general reduction of CNTF levels in perilesional regions [5]. We observed a modest reduction of *Cntf* mRNA levels in GCV-Nestin^TK^ mice sampled at day 31 (Fig. 5c). At this time point, also *Vegfa* mRNA levels were affected in GCV-Nestin^TK^ mice, although such reduction was a trend, not reaching the statistical significance (Fig. 5d). However, we also noticed that the simple administration of GCV to uninjured mice reduced *Vegfa* levels, suggesting that such alteration was probably relied on the pharmacological treatment (Fig. 5d). Overall, these data corroborate the idea that SC-NSC ablation affects the general expression of neurotrophic factors in the lesioned SC, which hampers endogenous mechanisms of tissue repair.

### The ablation of SC-NSCs modulates the perilesional inflammatory milieu

**Fig. 6:**
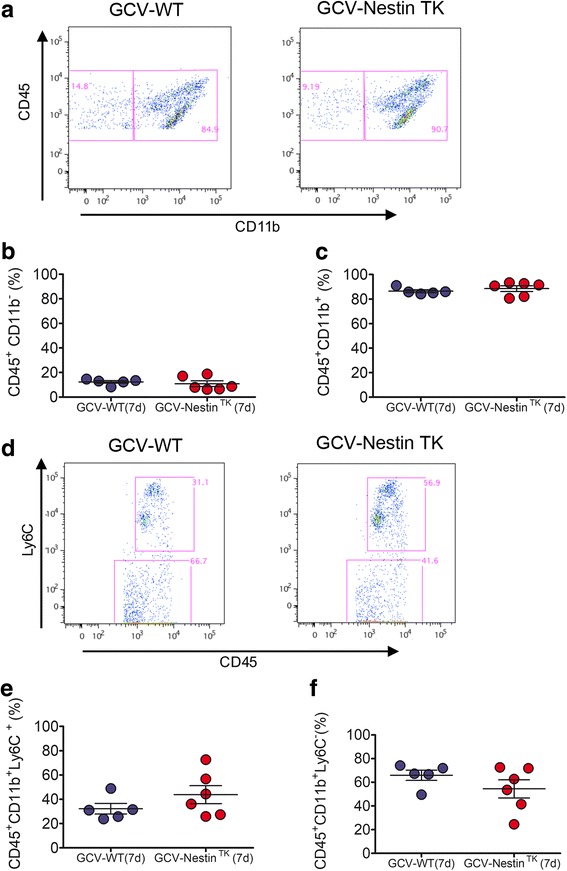
Modulation of the inflammatory response in GCV-Nestin^TK^ mice. Representative dot plots showing the gating strategy for the identification of lymphoid (CD45^+^ CD11b^-)^ and myeloid (CD45^+^ CD11b^+^) cells, gated on the CD45^+^ cell populations isolated from injured spinal cord at day 7 (**a**). Quantification of lymphoid (**b**) and myeloid cells (**c**)**.** Representative dot plots showing the gating strategy for the identification of macrophage lineage cells (CD45^+^CD11b^−^Ly6C^+^) and microglia cells (CD45^+^CD11b^−^Ly6C^+^) cells (**d**). Quantification of macrophage lineage cells (**e**) and microglial lineage cells (**f**). For all quantifications red dots are GCV-Nestin^TK^ mice while blue dots are GCV-WT mice, each dot represents a single animal. Horizontal bars indicate mean values ±S.E.M. p=

**Fig. 7:**
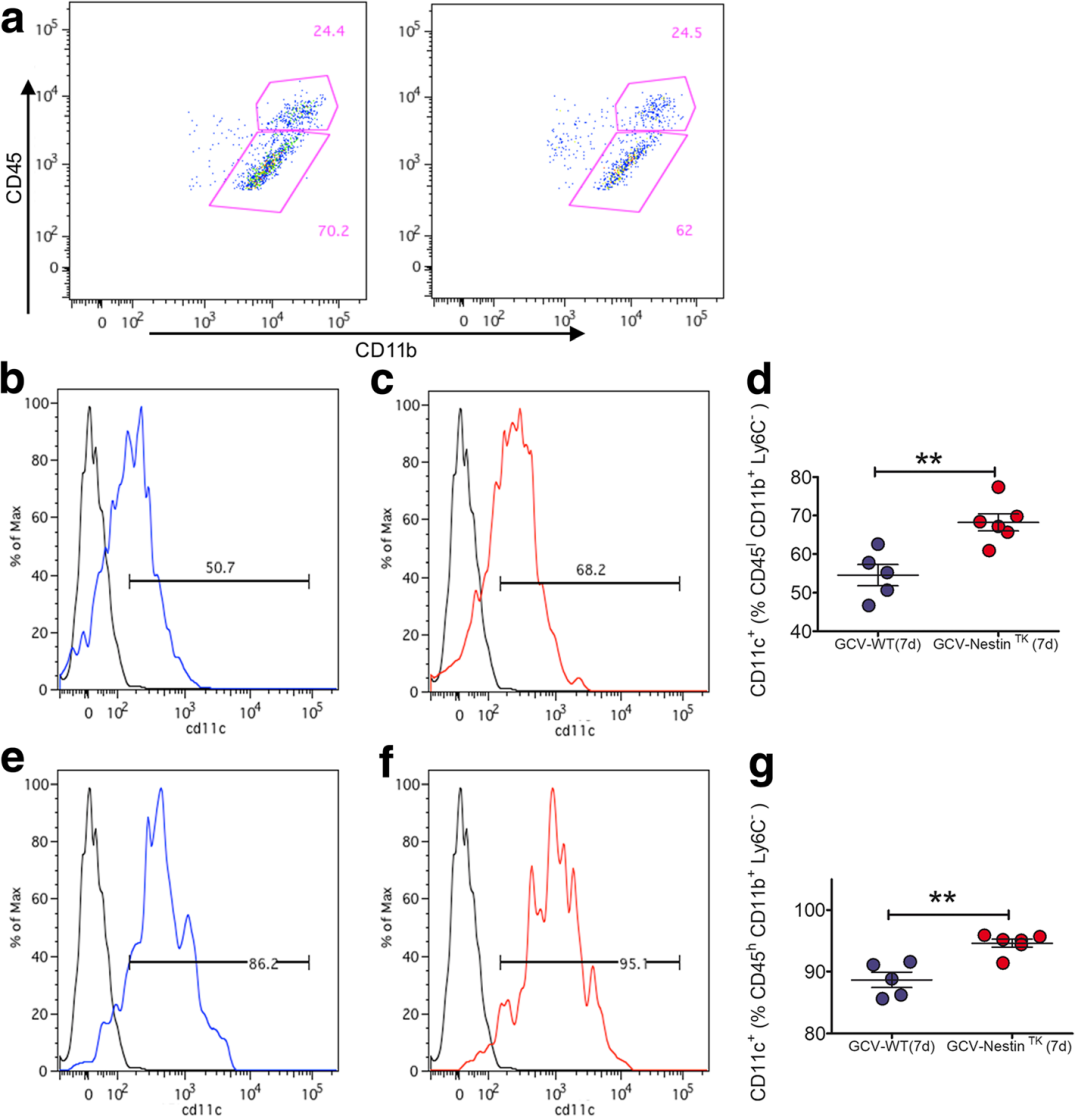
CD11c is increased in microglia of GCV-Nestin^TK^ mice. Representative dot plots showing the gating strategy for the identification of CD45^high^ and CD45^Int^ also expressing CD11b, gated on the CD45^+^ Ly6C^−^ cells (**a**). Quantifications of the expression level CD11c on CD45^I^CD11d^+^Ly6C^−^cells (**b**-**c**) and CD11c on CD45^h^CD11d^+^Ly6C^−^ cells (**e**-**g**). For all quantifications red dots are GCV-Nestin^TK^ mice while blue dots are GCV-WT mice, each dot represents a single animal. Unpaired t test, ** *p* < 0.01

Besides activation of microglia and astrocytes, a local inflammatory response involving blood-borne cells dominates the site of the injury [36, 41]. To find out the possible interplay between SC-NSCs and the inflammatory response, we analyzed the identity and the phenotype of inflammatory cells located at the injury site. We dissociated lesioned SCs from GCV-Nestin^TK^ and GCV-WT mice at day 7 and using flow cytometry we evaluated the distribution of different cell subpopulations that were taking part to the inflammatory response. The lack of SC-NSCs modulate neither percentages of bona fide lymphocytes (CD45^+^CD11b^−^), (Fig. 6a, b) nor percentages of CD45^+^CD11b^+^expressing cells (Fig. 6a, c). We next scored the cell morphology of activated myeloid cells analyzing sections labeled for Iba1^+^F4/80^+^ cells at day 18. Double positive cells were well ramified in both experimental groups when examined 3 mm far from the site of the injury (Additional file 7: Figure S5A, A’, C, and C’). At the site of the injury, Iba1^+^F4/80^+^ cells increased in their number, were amoeboid, and highly expressed F4/80. However, judging their cell morphology as well as their distribution around the lesion site, we did not find any relevant difference between GCV-WT and GCV-Nestin^TK^ mice (Additional file 7: Figure S5 B, B’, D, and D’). We next wanted to discriminate between activated microglia and infiltrating myeloid cells. We exploited the flow cytometry and the expression of the marker Ly6C to discriminate infiltrating CD45^+^CD11b^+^ cells (Fig. 6d), [42]. We observed that there was a slight decrease of microglia, which paralleled with a modest increment of CD45^+^CD11b^+^Ly6C^+^ cell number in GCV-Nestin^TK^ mice, although these differences were a trend that did not reach the statistical significance (Fig. 6d–f). We analyzed the activation profiles of microglia, scoring expression levels of CD11c in CD45^+^CD11b^+^Ly6C^−^ cells. On the basis of CD45 expression levels, we further subdivided this cell population in CD45 intermediate (CD45^I^) and CD45 high (CD45^h^) cells. Percentages of CD11c^+^ cells were significantly increased in CD11b^+^CD45^I^ as well as in CD11b^+^CD45^h^ cells of GCV-Nestin^TK^ mice (Fig. 7b–f). These results mirrored our published data, showing that SCI mice receiving an extra amount of NSCs by transplantation reduced CD11c expression levels in myeloid cells [4]. Since CD11c^+^ cells usually participate to the pro-inflammatory response secreting inflammatory cytokines [4], we next assayed mRNAs levels of some pro-inflammatory cues in GCV-Nestin^TK^ mice. At day 7, SCI mice lacking SC-NSCs significantly increased the expression of *TNFα* and *IL-1β* mRNA levels when compared with GCV-WT mice, (Additional file 8: Figure S6A–B). Similarly, *iNOS* and *IL-6* mRNA levels were increased in GCV-Nestin^TK^, although these differences were a trend not reaching the statistical significance (Additional file 8: Figure S6D). However, the elevation of *TNFα* and *IL-1β* mRNA was transient and at day 31 and we did not find any significant difference between control mice and mice lacking SC-NSCs (Additional file 8: Figure S6A–D). All in all, the ablation of SC-NSCs greatly impairs locomotion recovery in mice and increases the content of M1-like myeloid cells in the perilesional area.

## Discussion

The central canal of the adult SC contains SC-NSCs that undergo to functional activation in response to tissue damage [43]. The remarkably activation of SC-NSCs, well documented in mice receiving the SCI, involves cell proliferation and the secretion of diffusible molecules [11]. Blocking their proliferation capacity resulted in a reduction of spontaneous tissue recovery [5]. This is partially due to the innate ability of SC-NSC descendants to secrete growth factors that promote tissue recovery. To assess the role of endogenous SC-NSCs in the spontaneous recovery occurring in the injured SC, we performed the genetic ablation of Nestin-expressing SC-NSCs. Our approach is based on the concept that besides Vimentin, SC-NSCs also express Nestin, and transgenic mice carrying a TK suicide gene under the control of Nestin regulatory region can be used to ablate SC-NSCs [10, 19]. We initially characterized our transgenic mice, carrying a floxable GFP cassette before the TK gene, to demonstrate that they can be definitely used to trace and to target SC-NSCs. We confirmed that GFP^+^ cells deriving from Nestin ^flox^GFP^flox^-TK mice are able to generate neurospheres in vitro as well as that such cells can give rise to oligodendrocytes, astrocytes, and neurons upon growth factor withdrawal. A key aspect of the SC-NSCs response to tissue damage includes the cell division. In agreement with published data [5, 36], we also demonstrated that GFP^+^BrdU^+^ cells increased in the injured spinal cord of Nestin ^flox^GFP^flox^-TK mice. We observed that GFP^+^BrdU^+^ cells were mainly located anteriorly to the site of the lesion, suggesting that some signals can differentially regulate the SC-NSC cell proliferation during SCI. Using transgenic mice expressing the Cre^ERT2^ allele under the control of the *Nestin* promoter [25], we were able to fate mapping SC-NSC descendants in SCI mice. Starting from 2 weeks after the injury a considerable high number of YFP^+^ cells differentiated in astrocytes that mainly surrounded the injury site, while few of them remained undifferentiated. In addition to astrocytes, some SC-NSCs differentiated in Olig2^+^ cells, reinforcing the idea that SC-NSCs sustain the myelination of the injured SC. Considering that NG2^+^ progenitors increase rates of cell proliferation in response to SCI [36], we could speculate that some GFP^+^BrdU^+^ cells scored in the gray matter of SCI mice may belong to the oligodendrocytes precursors cell population.

Our knowledge about SC-NSCs in the SCI model is based on experimental approaches that inhibit their ability to enter the cell cycle or to migrate toward perilesional sites [5, 44]. In our experimental setting, we killed a cohort of proliferating Nestin^+^ cells. Such cells are bona fide slowly dividing NSCs that may be relevantly involved in tissue repair. Such approach significantly dampened the number of proliferating ependymal cells but not completely abolish the entire population of SC-NSCs. However, the net result of this manipulation was a substantial worsening of clinical outcomes in mice. Thus, despite the fact that proliferating Nestin^+^ cells are normally few in the healthy ependymal layer, their ablation resulted in a less responsiveness of the CNS to micro environmental changes occurring upon SCI and in turn to a worsening of the functional recovery.

Published results indicate that a reduction of SC-NSC cell proliferation affects the formation of a compact scar [5]. On the other hand, the inactivation of β1-integrin in SC-NSCs enhances astrocyte migration to the injury site and contributes to the scar formation [44]. Both manipulations are detrimental for mice and affect their locomotion, indicating that SC-NSCs regulate the formation of the scar and such aspect is relevant for repair processes [5, 44]. Unexpectedly, the ablation of proliferating SC-NSCs occurring in GCV-Nestin^TK^ mice worsened clinical outcomes in mice without modulating the size of the scar. Such difference could be explained considering that we transiently depleted the proliferating pool ependymal Nestin^+^ cells. In principle, other ependymal cells, as well as surviving Nestin^+^ cells, could participate to the injury response at later time points. Such cells could contribute to seal the injury, preventing an excessive enlargement of the damaged tissue, but they do not rescue the locomotion of mice.

We observed that demyelination was significantly increased in regions located anteriorly to the site of damage. Interestingly, this is the region of the SCI that is also featured by an intense cell proliferation of SC-NSCs, as we have shown in Nestin ^flox^GFP^flox^-TK mice. Thus, we can speculate that the depletion of these cells deeply influences the remyelination process, reducing the number of Olig2^+^ cells and\or affecting the release of neurotrophic factors in the injured tissue.

Interestingly, parallel to the lack of SC-NSCs, we observed increasing percentages of microglia/macrophages that express the CD11c marker. This result is in line with our previously published data showing that SCI mice receiving NSCs transplantation display a substantial restoration of locomotion that parallel a striking reduction of CD11c^+^CD206^−^ cells in SC [4]. We explained these results considering that transplanted NSCs can skew microglia/macrophages to acquire neuroprotective phenotype in the injured SC [4]. Here, we observed that mice lacking SC-NSCs increased percentages of CD11c expressing cells as well as the expression levels of pro-inflammatory cytokines. Thus, as previously described for transplanted NSCs, also resident SC-NSCs can modulate the activation of microglia/macrophages that occurs in response to tissue damaging [18, 45, 46].

## Conclusion

Comparing our results, raised on mice lacking endogenous SC-NSCs, with previous observations that have been done on NSCs transplanted mice [4], we can speculate that both cell types exert a significant immunomodulatory function in the injured CNS, once they are facing inflammation. Such observation is not trivial, since transplanted NSCs are expanded in vitro for weeks or even months before being transplanted. Nonetheless, in vitro manipulations do not obstacle NSCs to perform a modulation of the inflamed environment, once transplanted. Similarly, we could speculate that endogenous SC-NSCs can exert a substantial “bystander” effect in the damaged tissue, limiting inflammatory cascades arising from parenchymal microglia and/or CNS-infiltrating immune cells. If we consider that the degree of inflammation correlates with the severity of SCI outcomes, the identification of signals released by SC-NSCs ought to give us new strategies in the perspective of a therapeutic approach based on NSC transplantation or on fostering the beneficial functions of the endogenous NSCs.

## Additional files

Additional file 1: **Figure S1.** Characterization of the spinal cord ependymal canal of a Nestin ^flox^GFP^flox^-TK mouse. Representative confocal images showing immunofluorescences for GFP/GFAP (A), and GFP/Id1 (B) in the ependymal layer of a Nestin ^flox^GFP^flox^-TK mice (*n* = 3). Scale bar is 30 μm. (TIFF 1498 kb)

Additional file 2: **Figure S2.** Sorted GFP+ cells from Nestin ^flox^GFP^flox^-TK mice give rise to neurospheres. Panel *A* and *B* show the gating strategy for sorting GFP^+^ cells from SCs bulk cultures obtained from Nestin ^flox^GFP^flox^-TK mice. WT litters (*A*) were used to set up the gating strategy that we used to sort GFP^+^ cells (B). GFP^+^ cells were plated at the density of 8000 cells/cm^2^ and daily examined for the presence of neurospheres. Small spheres were observed after 3 days (C), while spheres with diameters larger than 100 μm were easily observed after 7 days (D). Scale bar 50 μm (*n* = 3 independent preparations). (TIFF 9717 kb)

Additional file 3: **Figure S3.** GCV treatment ablates proliferating SC-eNSCs in Nestin^TK^ mice. Panels *A* and *B* show representative confocal images of VIM (red) and Brdu (green) in the ependymal layer of GCV-WT (*A*) and GCV-Nestin^TK^ (*B*) mice (*n* = 3 for each group). Mice were sacrificed at the end of the GCV treatment. Quantifications (means ± S.E.M.) are shown in panel *C*. Two-way ANOVA followed by Bonferroni’s multiple Comparison test has been used to analyze data. ** *p* = 0.011 and *p* = 0.045 in thoracic and lumbar segments, respectively. Scale bar 20 μm. (TIFF 2754 kb)

Additional file 4: **Figure S4.** Catwalk assay on GCV-Nestin^TK^ mice. Histogram in panel *A*, shows locomotor functions of GCV-WT mice (blued dots, *n* = 6) and GCV-Nestin^TK^ mice (red dots, *n* = 6). Data are represented as Basso Mouse Scale (± S.E.M.) mean values of the locomotor score of each group. Statistical analysis in panel A has been done comparing at each time point GCV-WT and GCV-Nestin^TK^ mice. Day 3 *p* = 0.015; day 5 *p* = 0.00047; day 7 *p* = 0.00014; day 10 *p* = 0.00031; day 14 *p* = 0.00046; day 18 *p* = 0.00019. Footprint and timing view of a GCV-WT (*A*) and a GCV-Nestin^TK^ (*B*) mouse acquired by the Catwalk XT Gait Analysis System. RF (right front), LF (left front), LH (left hind), RH (right hind) paws. (TIFF 4588 kb)

Additional file 5: **Movie S1** Video of Catwalk gait analysis of a representative GCV- WT mouse. WT mice were treated with GCV, subjected to SCI and followed for locomotor recovery by BMS score when behavioral amelioration reached a plateau value mice, were subjected to Catwalk gait analysis. The video shows recording of a representative GCV-WT animal (BMS score = 3). Free ambulation along an illuminated glass plate in a darkened room has been recoded for 4.30 s. Video has been captured at day 18 after SCI. (AVI 139494 kb)

Additional file 6: **Movie S2** Video of Catwalk gait analysis of a representative GCV-Nestin^TK^ mouse. Nestin^TK^ mice were treated with GCV, subjected to SCI and followed for locomotor recovery by BMS score when behavioral amelioration reached a plateau value, mice were subjected to Catwalk gait analysis. The video shows recording of a representative GCV-Nestin^TK^ animal (BMS score = 1). Free ambulation along an illuminated glass plate in a darkened room has been recorded for 3.22 s. Video has been captured at day 18 after SCI. (AVI 104621 kb)

Additional file 7: **Figure S5.** macrophages/macrophages activation affects both GCV-WT and GCV-Nestin^TK^ mice. (*A*) Histological analysis of a SC region located 3 mm far from the injury site from a GCV-WT mice (18 days post injury). Microglia/macrophages are labeled for Iba1 and F4/80. Arrowhead indicates cells that are shown at high magnification in panel *A’*. Panel B shows the site of the injury in the SC of GCV-WT mouse. Arrowhead indicates cells that are shown at high magnification in panel *B′*. A representative section of the SC located 3 mm far from the site of the injury from a GCV-Nestin^TK^ mouse (18 days post injury) is shown in panel *C*. Arrowhead indicates cells that are shown at high magnification in panel *C′*. Panel *D* shows the site of the injury while the arrowhead indicates cells that are shown at high magnification in *D’* (*n* = 3 for each group). Scale bar 50 μm (TIFF 4124 kb)

Additional file 8: **Figure S6.** Upregulation of inflammatory cues in GCV-Nestin^TK^ mice. Real-time PCR analysis of pro-inflammatory genes (*A–D*) in T11–T13 spinal cord tissues at different time points after the injury induction. GCV-Nestin^TK^ mice (red bars) have a increased expression of pro-inflammatory genes after injury compared with control mice (blue bars). Values indicate mean fold changes ± S.E.M (*n* = 3–6 for each group). Comparisons were done using the *t* Student test: *TNFα*:* *p* = 0.021; *IL-1β*:* *p* = 0.046; *Vegfa*: *p* = 0.008. (TIFF 7997 kb)

## Abbreviations

BMS: Basso Mouse Scale
BrdU: 5-Bromo-2′-deoxyuridine
CNS: Central nervous system
GCV: Ganciclovir
NSC: Neural stem cell
SC: Spinal cord
SCI: Spinal cord injury
SC-NSCs: Spinal cord neural stem cells
SVZ: Sub-ventricular zone
TK: Thymidine kinase 1
VIM: Vimentin
WT: Wild type

## References

[1] Singh A (2014). Global prevalence and incidence of traumatic spinal cord injury. Clin Epidemiol.

[2] Kokaia Z (2012). Cross-talk between neural stem cells and immune cells: the key to better brain repair?. Nat Neurosci.

[3] Martino G (2011). Brain regeneration in physiology and pathology: the immune signature driving therapeutic plasticity of neural stem cells. Physiol Rev.

[4] Cusimano M (2012). Transplanted neural stem/precursor cells instruct phagocytes and reduce secondary tissue damage in the injured spinal cord. Brain.

[5] Sabelstrom H (2013). Resident neural stem cells restrict tissue damage and neuronal loss after spinal cord injury in mice. Science.

[6] Barnabe-Heider F, Frisen J (2008). Stem cells for spinal cord repair. Cell Stem Cell.

[7] Alvarez-Buylla A, Herrera DG, Wichterle H (2000). The subventricular zone: source of neuronal precursors for brain repair. Prog Brain Res.

[8] Alfaro-Cervello C (2012). Biciliated ependymal cell proliferation contributes to spinal cord growth. J Comp Neurol.

[9] Hamilton LK (2009). Cellular organization of the central canal ependymal zone, a niche of latent neural stem cells in the adult mammalian spinal cord. Neuroscience.

[10] Meletis K (2008). Spinal cord injury reveals multilineage differentiation of ependymal cells. PLoS Biol.

[11] Barnabe-Heider F (2010). Origin of new glial cells in intact and injured adult spinal cord. Cell Stem Cell.

[12] Popovich PG, Wei P, Stokes BT (1997). Cellular inflammatory response after spinal cord injury in Sprague-Dawley and Lewis rats. J Comp Neurol.

[13] Mawhinney LA (2012). Differential detection and distribution of microglial and hematogenous macrophage populations in the injured spinal cord of lys-EGFP-ki transgenic mice. J Neuropathol Exp Neurol.

[14] Kigerl KA (2009). Identification of two distinct macrophage subsets with divergent effects causing either neurotoxicity or regeneration in the injured mouse spinal cord. J Neurosci.

[15] Shechter R (2011). The glial scar-monocyte interplay: a pivotal resolution phase in spinal cord repair. PLoS One.

[16] Shechter R (2009). Infiltrating blood-derived macrophages are vital cells playing an anti-inflammatory role in recovery from spinal cord injury in mice. PLoS Med.

[17] Immig K (2015). CD11c-positive cells from brain, spleen, lung, and liver exhibit site-specific immune phenotypes and plastically adapt to new environments. Glia.

[18] Pluchino S (2005). Neurosphere-derived multipotent precursors promote neuroprotection by an immunomodulatory mechanism. Nature.

[19] Butti E (2012). Subventricular zone neural progenitors protect striatal neurons from glutamatergic excitotoxicity. Brain.

[20] Pluchino S (2009). Immune regulatory neural stem/precursor cells protect from central nervous system autoimmunity by restraining dendritic cell function. PLoS One.

[21] Pluchino S, Martino G (2005). The therapeutic use of stem cells for myelin repair in autoimmune demyelinating disorders. J Neurol Sci.

[22] Pluchino S (2003). Injection of adult neurospheres induces recovery in a chronic model of multiple sclerosis. Nature.

[23] Su H (2002). A targeted X-linked CMV-Cre line. Genesis.

[24] Kawaguchi A (2001). Nestin-EGFP transgenic mice: visualization of the self-renewal and multipotency of CNS stem cells. Mol Cell Neurosci.

[25] Imayoshi I (2008). Roles of continuous neurogenesis in the structural and functional integrity of the adult forebrain. Nat Neurosci.

[26] Soriano P (1999). Generalized lacZ expression with the ROSA26 Cre reporter strain. Nat Genet.

[27] Galvan MD (2008). Deficiency in complement C1q improves histological and functional locomotor outcome after spinal cord injury. J Neurosci.

[28] Cardona AE (2006). Isolation of murine microglial cells for RNA analysis or flow cytometry. Nat Protoc.

[29] Li X (2016). Regenerative potential of ependymal cells for spinal cord injuries over time. EBioMedicine.

[30] Zimmerman L (1994). Independent regulatory elements in the nestin gene direct transgene expression to neural stem cells or muscle precursors. Neuron.

[31] Nam HS, Benezra R (2009). High levels of Id1 expression define B1 type adult neural stem cells. Cell Stem Cell.

[32] Weiss S (1996). Is there a neural stem cell in the mammalian forebrain?. Trends Neurosci.

[33] Doetsch F, Garcia-Verdugo JM, Alvarez-Buylla A (1997). Cellular composition and three-dimensional organization of the subventricular germinal zone in the adult mammalian brain. J Neurosci.

[34] Lim DA, Alvarez-Buylla A (2014). Adult neural stem cells stake their ground. Trends Neurosci.

[35] Frederiksen K, McKay RD (1988). Proliferation and differentiation of rat neuroepithelial precursor cells in vivo. J Neurosci.

[36] Horky LL (2006). Fate of endogenous stem/progenitor cells following spinal cord injury. J Comp Neurol.

[37] Basso DM (2006). Basso mouse scale for locomotion detects differences in recovery after spinal cord injury in five common mouse strains. J Neurotrauma.

[38] Hayakawa K (2015). Intrathecal injection of a therapeutic gene-containing polyplex to treat spinal cord injury. J Control Release.

[39] Laterza C (2013). iPSC-derived neural precursors exert a neuroprotective role in immune-mediated demyelination via the secretion of LIF. Nat Commun.

[40] Bacigaluppi M (2016). Neural stem cell transplantation induces stroke recovery by upregulating glutamate transporter GLT-1 in astrocytes. J Neurosci.

[41] Popovich PG, Hickey WF (2001). Bone marrow chimeric rats reveal the unique distribution of resident and recruited macrophages in the contused rat spinal cord. J Neuropathol Exp Neurol.

[42] Greter M, Lelios I, Croxford AL (2015). Microglia versus myeloid cell nomenclature during brain inflammation. Front Immunol.

[43] Johansson CB (1999). Identification of a neural stem cell in the adult mammalian central nervous system. Cell.

[44] North HA (2015). beta1-integrin alters ependymal stem cell BMP receptor localization and attenuates astrogliosis after spinal cord injury. J Neurosci.

[45] Butovsky O (2006). Microglia activated by IL-4 or IFN-gamma differentially induce neurogenesis and oligodendrogenesis from adult stem/progenitor cells. Mol Cell Neurosci.

[46] Pluchino S (2008). Persistent inflammation alters the function of the endogenous brain stem cell compartment. Brain.

