# Supplementary figures and images for: Selective killing of spinal cord neural stem cells impairs locomotor recovery in a mouse model of spinal cord injury
Source: J Neuroinflammation. 2018 Feb 23;15:58. doi: 10.1186/s12974-018-1085-9 (PMC5824446; doi:10.1186/s12974-018-1085-9)

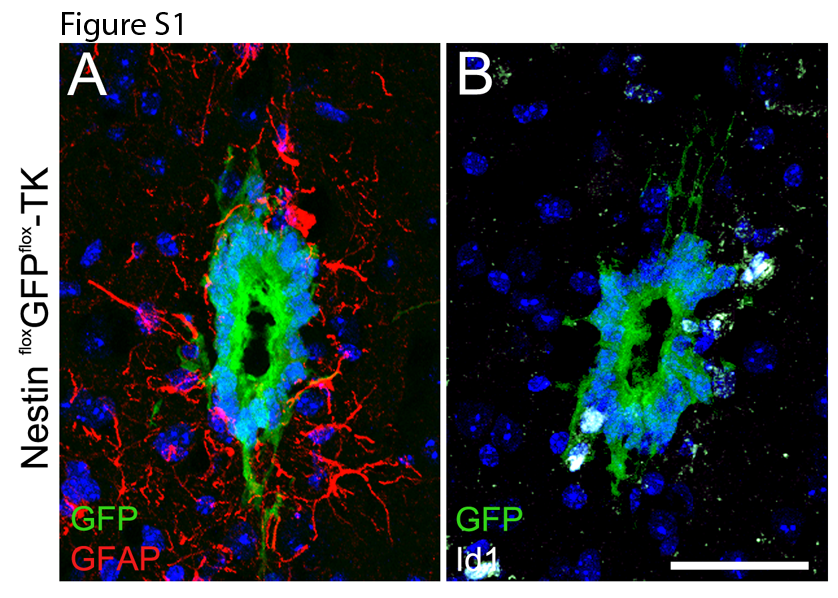

Supplement: Supplementary file 1 — Figure S1. Characterization of the spinal cord ependymal canal of a Nestin floxGFPflox-TK mouse. Representative confocal images showing immunofluorescences for GFP/GFAP (A), and GFP/Id1 (B) in the ependymal layer of a Nestin floxGFPflox-TK mice (n = 3). Scale bar is 30 μm. (TIFF 1498 kb) [file 12974_2018_1085_MOESM1_ESM.tif]

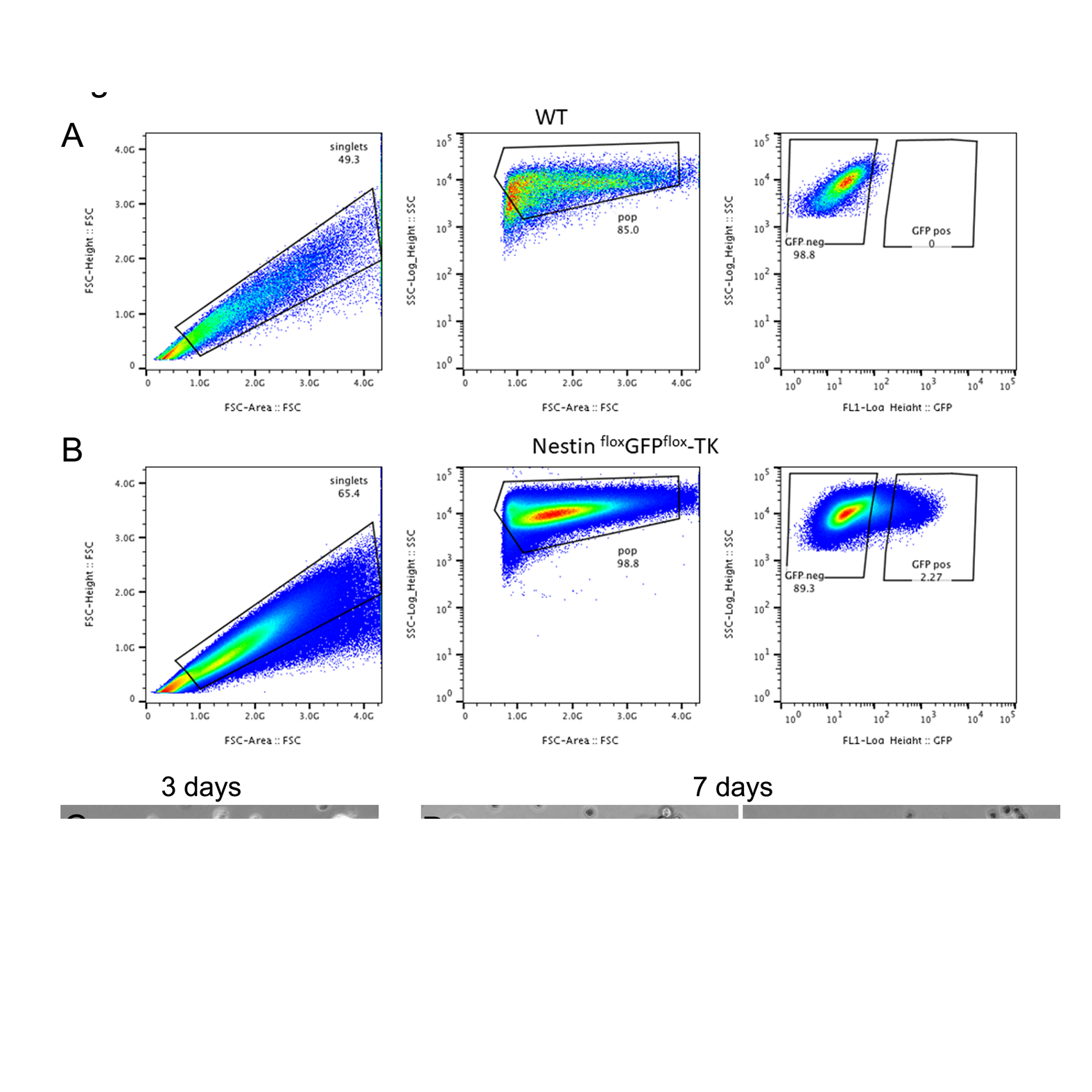

Supplement: Supplementary file 2 — Figure S2. Sorted GFP+ cells from Nestin floxGFPflox-TK mice give rise to neurospheres. Panel A and B show the gating strategy for sorting GFP+ cells from SCs bulk cultures obtained from Nestin floxGFPflox-TK mice. WT litters (A) were used to set up the gating strategy that we used to sort GFP+ cells (B). GFP+ cells were plated at the density of 8000 cells/cm2 and daily examined for the presence of neurospheres. Small spheres were observed after 3 days (C), while spheres with diameters larger than 100 μm were easily observed after 7 days (D). Scale bar 50 μm (n = 3 independent preparations). (TIFF 9717 kb) [file 12974_2018_1085_MOESM2_ESM.tif]

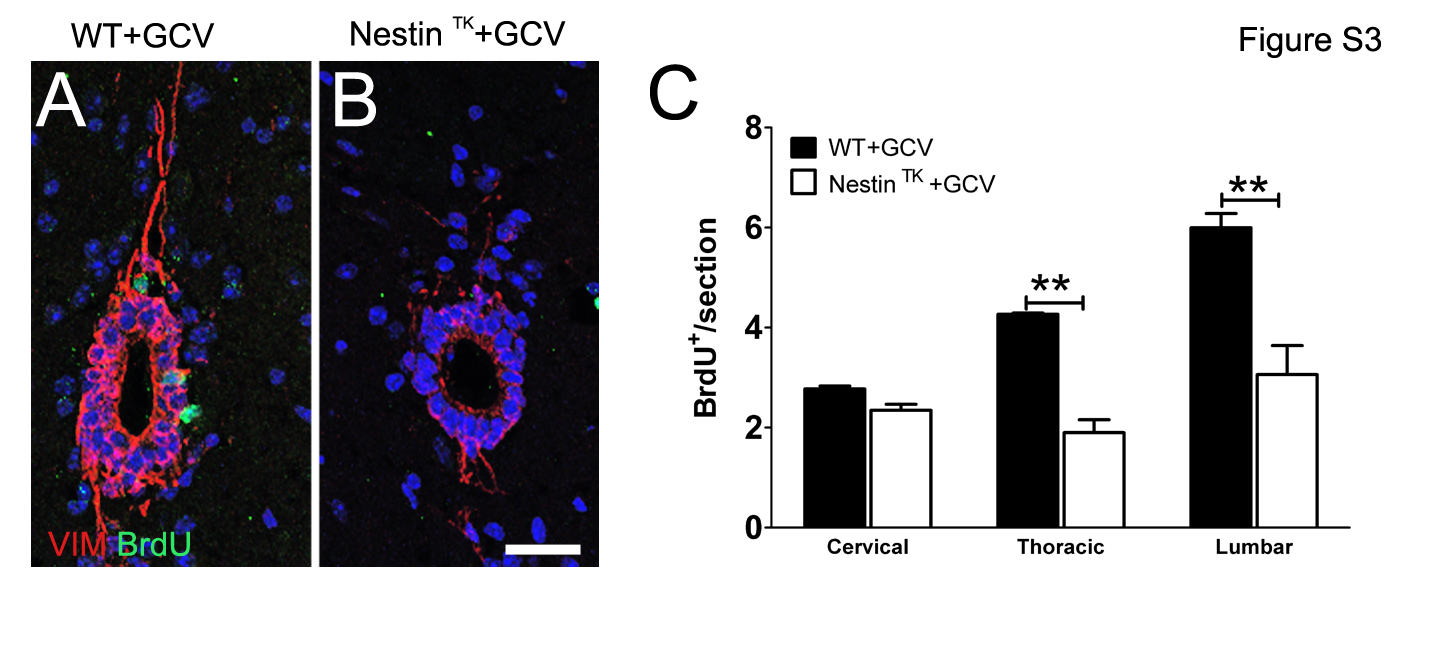

Supplement: Supplementary file 3 — Figure S3. GCV treatment ablates proliferating SC-eNSCs in NestinTK mice. Panels A and B show representative confocal images of VIM (red) and Brdu (green) in the ependymal layer of GCV-WT (A) and GCV-NestinTK (B) mice (n = 3 for each group). Mice were sacrificed at the end of the GCV treatment. Quantifications (means ± S.E.M.) are shown in panel C. Two-way ANOVA followed by Bonferroni’s multiple Comparison test has been used to analyze data. ** p = 0.011 and p = 0.045 in thoracic and lumbar segments, respectively. Scale bar 20 μm. (TIFF 2754 kb) [file 12974_2018_1085_MOESM3_ESM.tif]

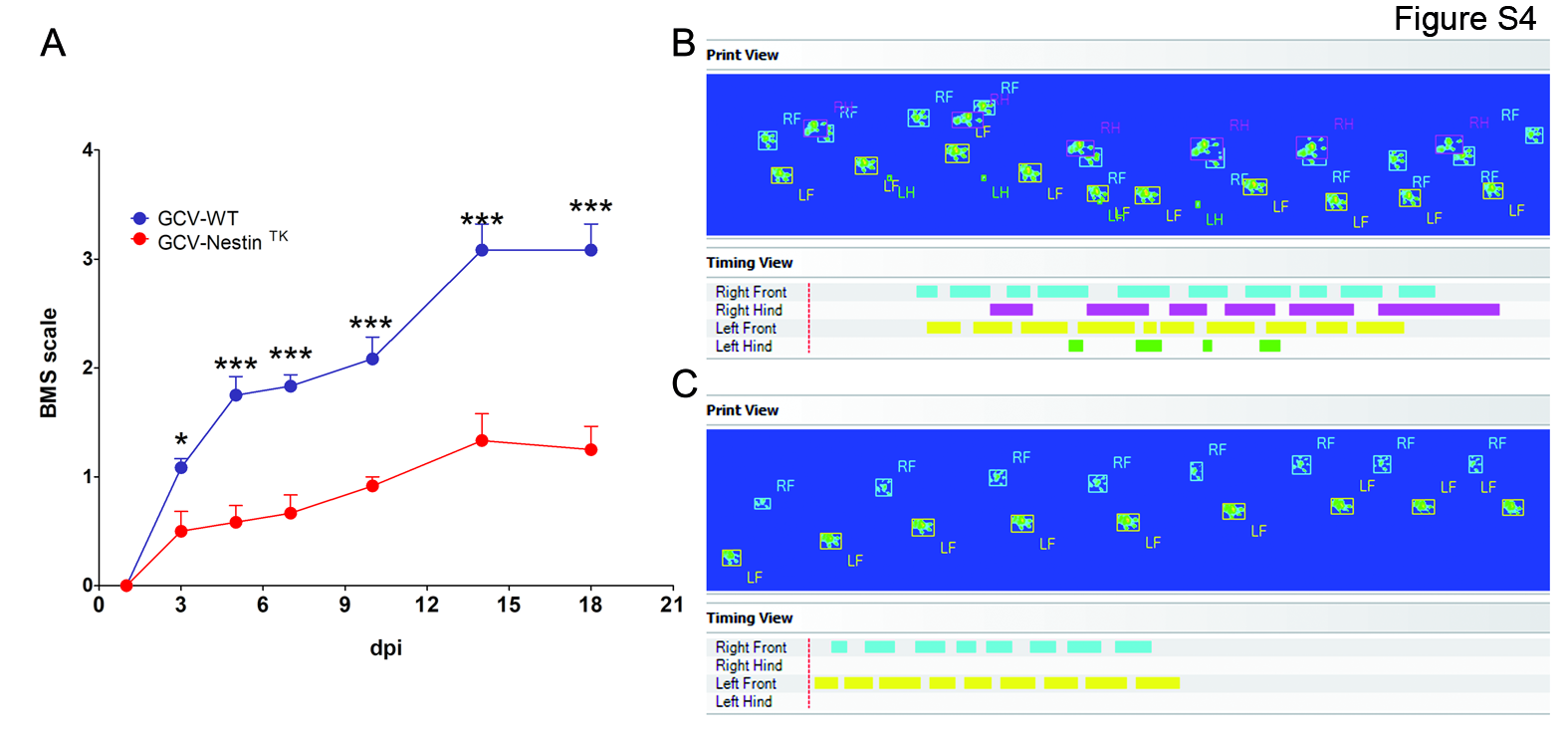

Supplement: Supplementary file 4 — Figure S4. Catwalk assay on GCV-NestinTK mice. Histogram in panel A, shows locomotor functions of GCV-WT mice (blued dots, n = 6) and GCV-NestinTK mice (red dots, n = 6). Data are represented as Basso Mouse Scale (± S.E.M.) mean values of the locomotor score of each group. Statistical analysis in panel A has been done comparing at each time point GCV-WT and GCV-NestinTK mice. Day 3 p = 0.015; day 5 p = 0.00047; day 7 p = 0.00014; day 10 p = 0.00031; day 14 p = 0.00046; day 18 p = 0.00019. Footprint and timing view of a GCV-WT (A) and a GCV-NestinTK (B) mouse acquired by the Catwalk XT Gait Analysis System. RF (right front), LF (left front), LH (left hind), RH (right hind) paws. (TIFF 4588 kb) [file 12974_2018_1085_MOESM4_ESM.tif]

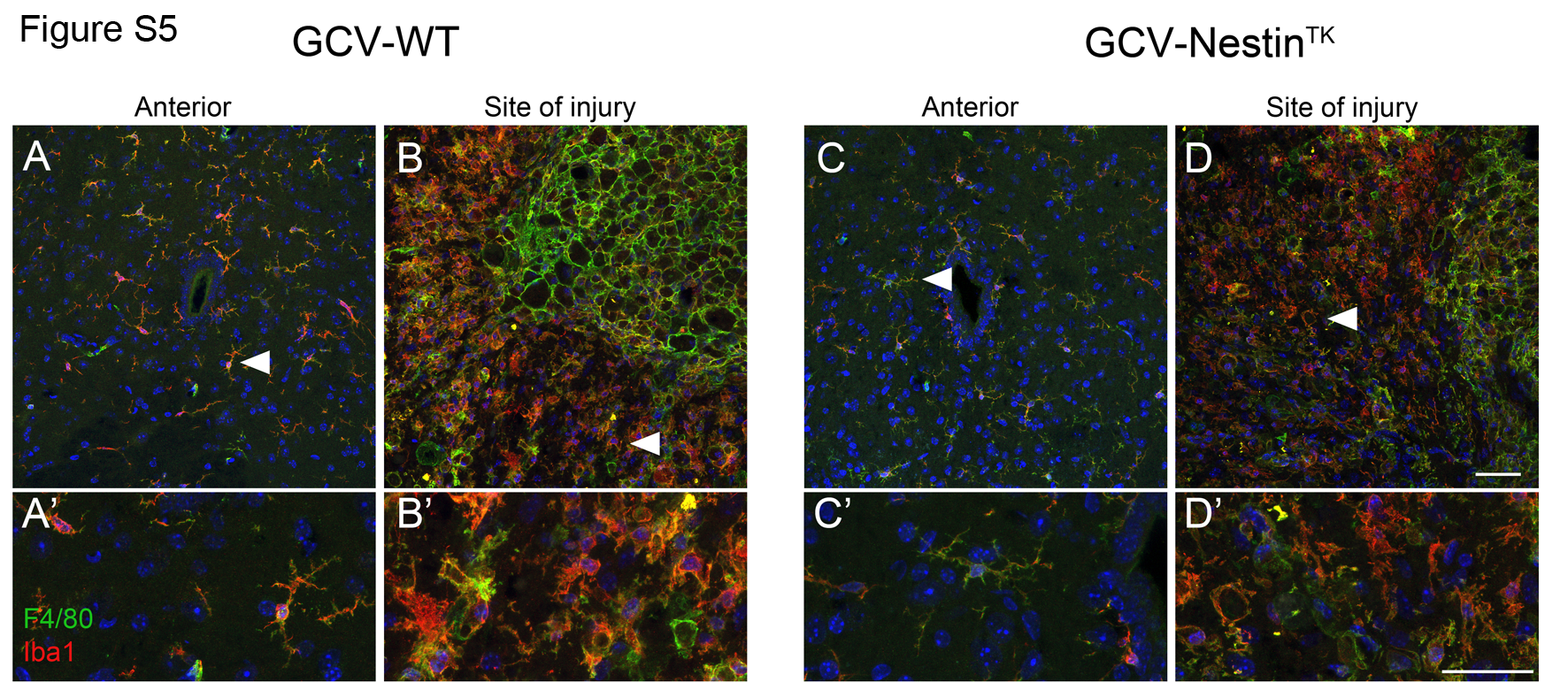

Supplement: Supplementary file 7 — Figure S5. macrophages/macrophages activation affects both GCV-WT and GCV-NestinTK mice. (A) Histological analysis of a SC region located 3 mm far from the injury site from a GCV-WT mice (18 days post injury). Microglia/macrophages are labeled for Iba1 and F4/80. Arrowhead indicates cells that are shown at high magnification in panel A’. Panel B shows the site of the injury in the SC of GCV-WT mouse. Arrowhead indicates cells that are shown at high magnification in panel B′. A representative section of the SC located 3 mm far from the site of the injury from a GCV-NestinTK mouse (18 days post injury) is shown in panel C. Arrowhead indicates cells that are shown at high magnification in panel C′. Panel D shows the site of the injury while the arrowhead indicates cells that are shown at high magnification in D’ (n = 3 for each group). Scale bar 50 μm (TIFF 4124 kb) [file 12974_2018_1085_MOESM7_ESM.tif]

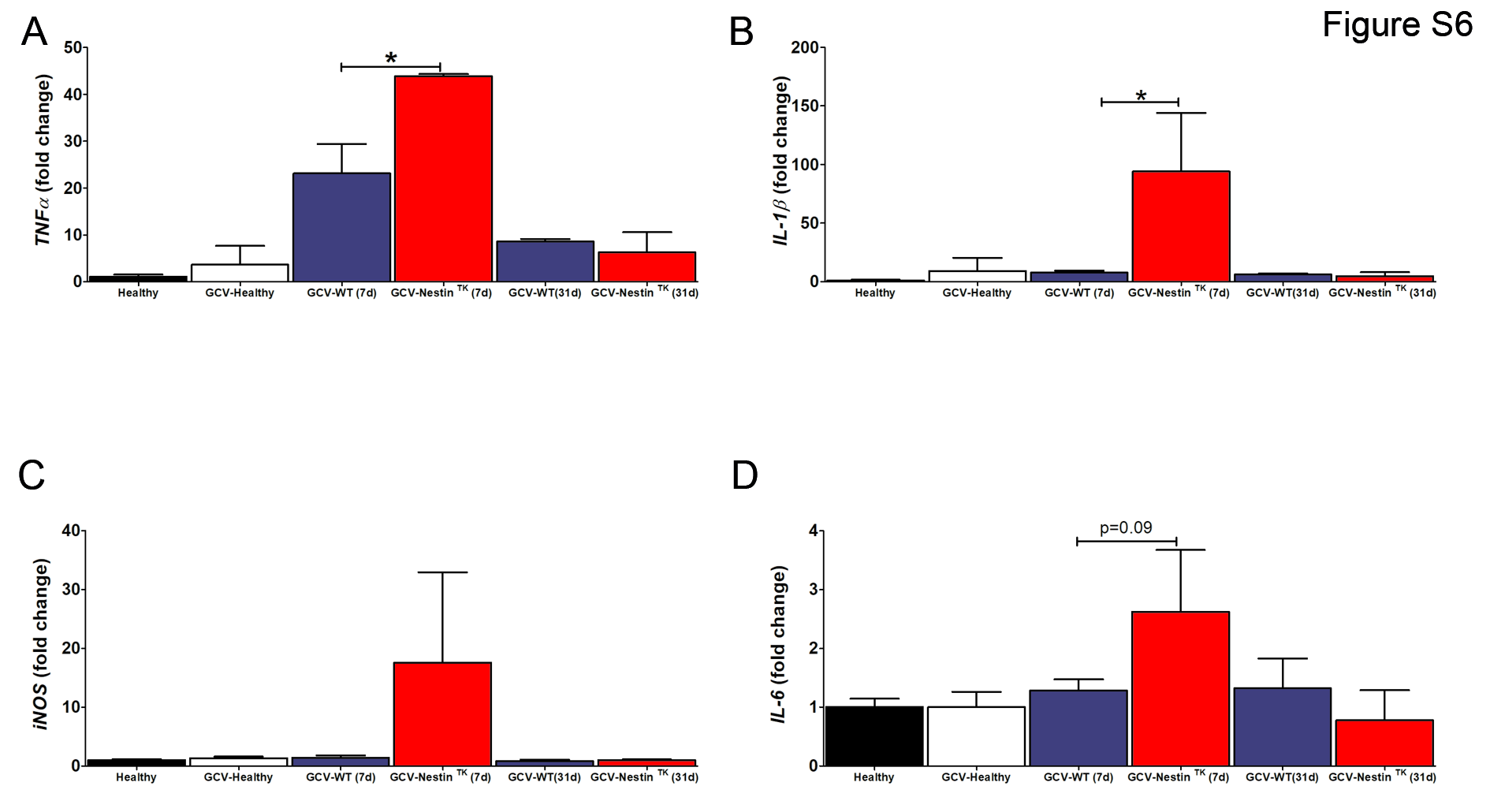

Supplement: Supplementary file 8 — Figure S6. Upregulation of inflammatory cues in GCV-NestinTK mice. Real-time PCR analysis of pro-inflammatory genes (A–D) in T11–T13 spinal cord tissues at different time points after the injury induction. GCV-NestinTK mice (red bars) have a increased expression of pro-inflammatory genes after injury compared with control mice (blue bars). Values indicate mean fold changes ± S.E.M (n = 3–6 for each group). Comparisons were done using the t Student test: TNFα:* p = 0.021; IL-1β:* p = 0.046; Vegfa: p = 0.008. (TIFF 7997 kb) [file 12974_2018_1085_MOESM8_ESM.tif]
